# The clinical characteristics of patients with asthma exposed to different environmental risk factors: A cross‐sectional study

**DOI:** 10.1002/iid3.923

**Published:** 2023-06-28

**Authors:** Laiheng Luo

**Affiliations:** ^1^ Department of Respiratory Medicine Jiangxi Province Hospital of Integrated Chinese and Western Medicine Nanchang Jiangxi China

**Keywords:** asthma, biomass, occupational exposure, smoking

## Abstract

**Background:**

Smoking, biomass, and occupational exposure are the main environmental risk factors for asthma. The purpose of this study was to analyze the clinical characteristics of exposure to these risk factors in patients with asthma.

**Methods:**

This cross‐sectional study enrolled patients with asthma from an outpatient department according to the Global Initiative for Asthma. Demographics, forced expiratory volume in 1 s (FEV1), FEV1%pred, FEV1/forced vital capacity (FVC), laboratory tests, asthma control test (ACT), asthma control questionnaire (ACQ) scores, and the inhaled corticosteroid (ICS) dose were recorded. A generalized linear mixed model was used to adjust for potential confounders.

**Results:**

A total of 492 patients with asthma were included in this study. Of these patients, 13.0% were current smokers, 9.6% were former smokers, and 77.4% were never smokers. Compared with never smokers, the current and former smokers had a longer duration of asthma; lower ACT scores, FEV1, FEV1%pred, and FEV1/FVC; and higher ACQ scores, IgE, FeNO, blood eosinophils, and ICS dose (*p* < .05). In addition, the patients exposed to biomass alone were older; had higher exacerbation in the past year; a longer duration of asthma; and lower FEV1, FEV1%pred, FEV1/FVC, IgE, and FeNO compared with smoking or occupational exposure alone. Compared with smoking exposure alone, patients with occupational exposure alone had a longer duration of asthma and lower FEV1, FEV1%pred, FVC, IgE, FeNO, and ICS dose (*p* < .05).

**Conclusions:**

There are significant differences in the clinical characteristics of patients with asthma depending on the smoking status. In addition, significant differences also observed among smoking, biomass, and occupational exposure.

## Graphical abstract

There were significant clinical differences among smoking, biomass, and occupational exposure.

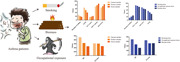

## 1 INTRODUCTION

Asthma is a highly heterogeneous chronic respiratory inflammatory disease characterized by wheezing, coughing, and chest tightness. Asthma has a high morbidity and affects about 350 million people worldwide.^1^ Therefore, treatment and prevention are urgent.

Environmental risk factors including smoking, biomass, and occupational exposure are adversely associated with asthma.^2^ Smoking is one of the preventable factors of asthma: around half the adult patients with asthma are current or former smokers.^3^ Studies have shown that long‐term smoking significantly reduces the sensitivity of patients with asthma to inhaled corticosteroids (ICS) and causes poor outcomes.^4^, ^5^ In addition, exposure to cigarette smoke could induce the release of proinflammatory mediators by activated neutrophils, macrophages, and T cells to cause airway inflammation and to promote the progression of asthma. Compared with never smokers, exposure to cigarette smoke in patients with asthma is associated with the recruitment, activation, and altered function of macrophages, natural killer cells, and T and B cells.^3^

Biomass including wood, charcoal, dried animal dung, and agricultural residues for cooking are the leading environmental risk factors for asthma in developing countries.^6^ Several studies have found that the use of biomass for cooking is associated with an increased risk of severe asthma symptoms.^7^ In addition, a study showed that biomass‐derived particulate matter could cause immune disorders and inflammation to aggravate asthma.^8^

Occupational exposure is another environmental risk factor for asthma.^9^ An estimated 5%−20% of new cases of adult‐onset asthma can be attributed to occupational exposure.^10^ In addition, studies have shown that persistent occupational exposure is associated with worse asthma outcomes.^11^ Compared with healthy controls, disease‐related immune functions in blood cells, including leukocyte migration, inflammatory responses, and decreased expression of upstream cytokines such as tumor necrosis factor and interferon gamma, are suppressed in patients who develop asthma from occupational exposure.^12^

Currently, numerous studies are focus on the diagnosis and pathogenesis of asthma caused by a single risk factor. However, there are no studies that have compared the clinical characteristics of asthma caused by different risk factors (i.e., smoking, biomass, and occupational exposure). Therefore, we investigated and compared the clinical characteristics of patients with asthma exposed to different environmental risk factors.

## 2 PATIENTS AND METHODS

### 2.1 Study participants

This was a cross‐sectional study. All subjects were from the outpatient department of the Jiangxi Hospital of Integrated Traditional Chinese and Western Medicine between January 2020 and June 2022. Asthma was diagnosed according to the Global Initiative for Asthma (GINA) guidelines, with bronchodilation forced expiratory volume in 1 s (FEV1) change >200 mL and 12%; positive bronchial stimulation test; and symptoms of asthma (including wheezing, difficulty breathing, chest tightness, or coughing).^13^ Patients with lung cancer, pneumonia; bronchiectasis; tuberculosis; and severe heart, liver, or kidney disease were excluded.

This study was conducted in accordance with the Declaration of Helsinki and approved by the Ethics Committee of the Jiangxi Province Hospital of Integrated Chinese and Western Medicine (Number: 202301). All patients provided their informed consent.

### 2.2 Data collection

Data including age; sex; education level; body mass index (BMI); smoking status; FEV1; FEV1%pred; FEV1/forced vital capacity (FVC); asthma control test (ACT); and asthma control questionnaire (ACQ) scores; exacerbations in the past year; laboratory tests including IgE, FeNO, and blood eosinophils; and ICS dose were collected at the patient's first visit.

### 2.3 Variable definition

A current smoker has had smoking exposure of ≥10 pack/years, while a former smoker has had ≥10 pack/years but had not smoked for more than 6 months. A never smoker had never smoked or had smoked fewer than 100 cigarettes in their lifetime.^14^ Biomass exposure was defined as using biomass fuels (wood, grass, charcoal, and crop residues) for cooking or heating at least 2 h per day for at least 1 year. Occupational exposure was defined as exposure to dust, gases/fumes, insecticides, chemical substances, paints, and metals at work for at least 8 h per day for more than 1 year.^15^ The ACQ consists of seven items, each scored from 0 to 6. In this study, the ACQ scores are the average of the seven items.^16^

### 2.4 Study procedures

The patients were divided into three groups according to the smoking status: current smoker, former smoker, and never smoker. The following criteria were used to distinguish the subgroups: smoking alone group, patients had only been exposed to cigarette smoke, including current and former smokers; biomass alone, patients had only been exposed to biomass; occupational exposure alone, patients had only been subjected to occupational exposure; and never smoking alone, patients had not been subjected to cigarette smoke, biomass, or occupational exposure.

### 2.5 Statistical analysis

SPSS 25.0 (IBM) was used for statistical analysis of the data. Continuous variables are expressed as mean ± standard deviation or median and interquartile range. Continuous variables with a normal distribution and homogeneous variances were analyzed with analysis of variance; otherwise, nonparametric tests were used. The *χ* ^2^ test or Fisher's exact test was used to analyze categorical variables. A logistic regression was used to determine the relative factors for smoking cessation and calculate the adjusted odds ratio (aOR) and adjusted 95% confidence interval (a95% CI). A generalized linear mixed model was generated to control for potential confounders. *p* < .05 was considered to be statistically significant.

## 3 RESULTS

### 3.1 The clinical characteristics in the different smoking status groups

**Figure 1:**
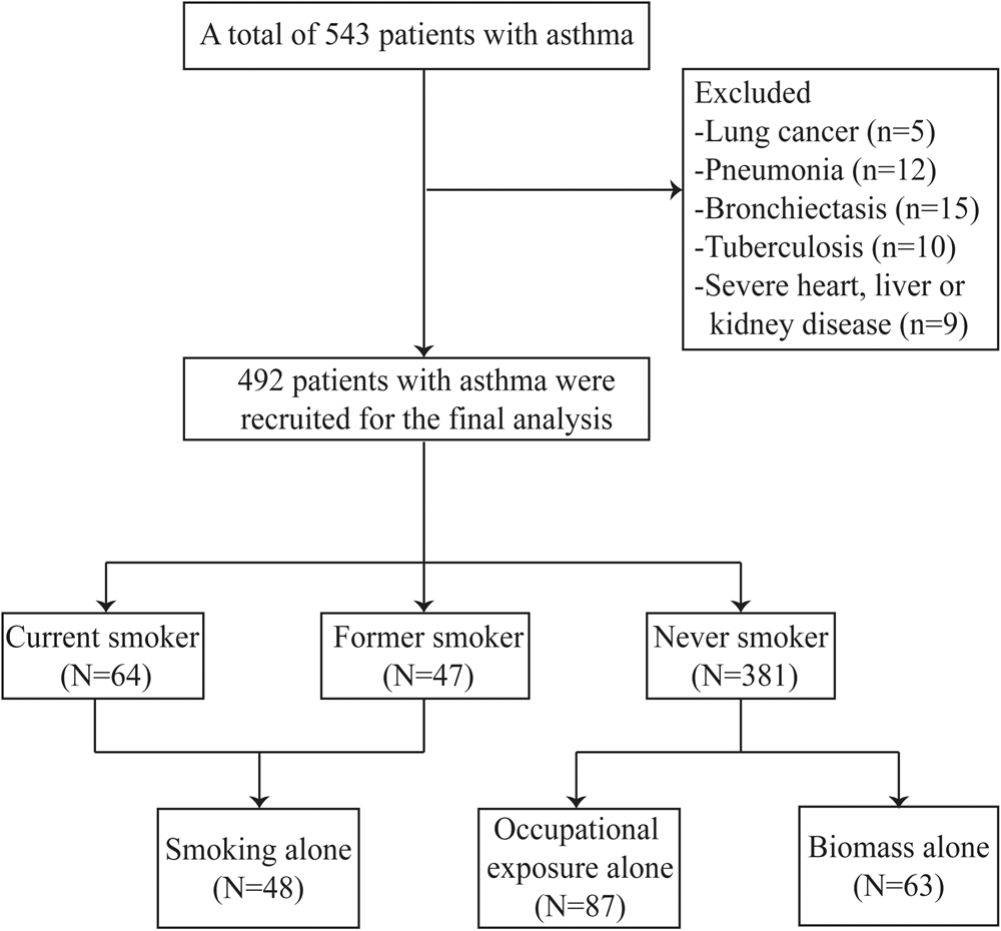
Flow chart of the study.

We included a total of 492 patients with asthma in this study (Figure 1). The mean age was 49.9 ± 13.2 years. Female accounted for 62.8% of the subjects. Of these patients, 77.4% were never smokers, 9.6% were former smokers, and 13.0% were current smokers (Table 1).

**Table 1:** The clinical characteristics in total asthma patients.

Variables	Total (*n* = 492)
Age (years), (mean ± SD)	49.9 ± 13.2
Sex, *n* (%)	
Male	183 (37.2)
Female	309 (62.8)
BMI (kg/m^2^), (mean ± SD)	24.0 ± 3.6
Education, *n* (%)	
Under junior high school	297 (60.4)
Over high school	195 (39.6)
Smoking (pack/year)	30 (12.5−50)
Smoking status, *n* (%)	
Never smoker	381 (77.4)
Former smoker	47 (9.6)
Current smoker	64 (13.0)
Biomass, *n* (%)	
Yes	108 (22.0)
No	384 (78.0)
Occupational exposure, *n* (%)	
Yes	163 (33.1)
No	329 (66.9)
Duration of asthma (years), (mean ± SD)	17.8 ± 10.2
Pulmonary function, (mean ± SD)	
FEV1	2.5 ± 0.7
FEV1%pred	80.3 ± 23.3
FEV1/FVC	73.9 ± 12.3
ACT, (mean ± SD)	17.6 ± 4.7
ACQ, (mean ± SD)	1.1 ± 0.7
Asthma control, *n* (%)	
Well controlled	77 (15.7)
Partially controlled	160 (32.5)
Uncontrolled	255 (51.8)
Exacerbations in the past year, (median, IQR)	0 (0−1)
Exacerbations in the past year, *n* (%)	
0	336 (68.3)
≥1	156 (31.7)
Biochemical indexes, (median, IQR)	
IgE (mg/L)	335.6 (113.0−760.2)
FeNO (ppb)	52.0 (22.0−64.0)
Blood eosinophils (×10^9^)	0.4 (0.2−0.7)
Treatment	
ICS dose (μg/day), (mean ± SD)	251.9 ± 140.2

Abbreviations: ACQ, asthma control questionnaire; ACT, asthma control test; BMI, body mass index; FeNO, fractionated exhaled nitric oxide; FEV1, forced expiratory volume in one second; FVC, forced vital capacity; ICS, inhaled corticosteroids; IQR, interquartile range; SD, standard deviation.

As shown in Table 2, the current and former smokers had a longer duration of asthma; lower ACT scores, FEV1, FEV1%pred, and FVC; and higher ACQ scores, IgE, FeNO, blood eosinophils, and ICS dose compared with never smokers (*p* < .05). Compared with former smokers, current smokers had a a longer duration of asthma; lower ACT scores, FEV1, FEV1%pred, and FVC; higher ACQ scores, IgE, FeNO, blood eosinophils, and number of exacerbations in the past year (*p* < .05).

**Table 2:** The clinical characteristics in different smoking status groups of asthma patients.

Variables	Current smoker	Former smoker	Never smoker	*p* Values
	(*n* = 64)	(*n* = 47)	(*n* = 381)	
Age (years), (mean ± SD)	47.6 ± 13.0^a^	57.6 ± 10.9	49.4 ± 13.1	**<.001**
Sex, *n* (%)				**<.001**
Male	59 (92.2)^b^	47 (100.0)^b^	77 (20.2)	
Female	5 (7.8)^b^	0 (0.0)^b^	304 (79.8)	
BMI (kg/m^2^), (mean ± SD)	24.4 ± 3.1	24.9 ± 3.4	23.8 ± 3.7	.128
Education, *n* (%)				.779
Under junior high school	41 (64.1)	29 (61.7)	227 (59.6)	
Over high school	23 (35.9)	18 (38.3)	154 (40.4)	
Biomass, *n* (%)				.609
Yes	11 (17.2)	11 (23.4)	86 (22.6)	
No	53 (82.8)	36 (76.6)	295 (77.4)	
Occupational exposure, *n* (%)				**.001**
Yes	31 (48.4)^b^	22 (46.8)^b^	110 (28.9)	
No	33 (51.6)^b^	25 (53.2)^b^	271 (71.1)	
Duration of asthma (years), (mean ± SD)	25.8 ± 9.2^a^^,^ b	19.5 ± 11.1^b^	16.7 ± 9.5	**<.001**
Pulmonary function, (mean ± SD)				
FEV1	1.9 ± 0.7^a^^,^ b	2.6 ± 0.8^b^	3.2 ± 0.9	**<.001**
FEV1%pred	70.5 ± 22.1^a^^,^ b	77.4 ± 22.5^b^	85.5 ± 23.1	**<.001**
FEV1/FVC	65.2 ± 12.4^a^^,^ b	72.6 ± 12.1^b^	82.7 ± 12.3	**<.001**
ACT, (mean ± SD)	14.7 ± 4.2^a^^,^ b	17.4 ± 4.7^b^	20.9 ± 4.8	**<.001**
ACQ, (mean ± SD)	1.2 ± 0.7^a^^,^ b	1.1 ± 0.7^b^	1.0 ± 0.6	**<.001**
Asthma control, *n* (%)				**.009**
Well controlled	2 (3.1)^b^	6 (12.8)	69 (18.1)	
Partially controlled	18 (28.1)	14 (29.8)	128 (33.6)	
Uncontrolled	44 (68.8)^b^	27 (57.4)	184 (48.3)	
Exacerbations in the past year, (median, IQR)	1 (0−1)^a^^,^ b	0 (0−1)	0 (0−1)	**.003**
Exacerbations in the past year, *n* (%)				**.008**
0	34 (53.1)^a^^,^ b	37 (78.7)	265 (69.6)	
≥1	30 (46.9)^a^^,^ b	10 (21.3)	116 (30.4)	
Biochemical indexes, (median, IQR)				
IgE (mg/L)	460.2 (215.7−1102.0)^a^^,^ b	316.2 (164.0−450.4)^b^	232.9 (120.5−453.0)	**<.001**
FeNO (ppb)	65 (20.8−64.0)^a^^,^ b	52.0 (35.5−65.0)^b^	39.0 (22.0−63.0)	**<.001**
Blood eosinophils (×10^9^)	0.6 (0.3−0.8)^a^^,^ b	0.4 (0.2−0.5)^b^	0.2 (0.1−0.4)	**.002**
Treatment				
ICS dose (μg/day), (mean ± SD)	305.5 ± 132.9^b^	300. 2 ± 152.4^b^	237.0 ± 136.7	**<.001**

*Note*: The bold *p* values indicate statistical significance.

Abbreviations: ACQ, asthma control questionnaire; ACT, asthma control test; BMI, body mass index; FeNO, fractionated exhaled nitric oxide; FEV1, forced expiratory volume in one second; FVC, forced vital capacity; ICS, inhaled corticosteroids; IQR, interquartile range; SD, standard deviation.

a *p* < .05 versus former smoker group;

^b^ *p* < .05 versus never smoker group.

### 3.2 Multivariate logistic regression of factors associated with smoking cessation in patients with asthma

After adjusting for FEV1%pred, FEV1/FVC, ACQ scores, and duration of asthma, logistic regression revealed that age (aOR = 1.121, a95% CI = 1.099−1.445, *p* = .011), over high school education level (aOR = 1.595, a95% CI = 1.358−1.988, *p* = .025), well controlled asthma (aOR = 7.006, a95% CI = 1.861−26.378, *p* = .004), and ACT scores (aOR = 1.788, a95% CI = 1.526−1.977, *p* < .001) were positively associated with smoking cessation. However, smoking (pack/years) (aOR = 0.666, a95% CI = 0.589−0.805, *p* = .003) and FEV1 (aOR = 0.701, a95% CI = 0.526−0.977, *p* < .001) were negatively associated with smoking cessation (Table 3).

**Table 3:** Multivariate analysis of relative factors for smoking cessation of asthma patients.

Variables	Univariate			Multivariate		
	OR	95% CI	*p* Values	aOR	a95% CI	a*p* Values
Age	1.120	1.098−1.443	**.019**	1.121	1.099−1.445	**.011**
Sex						
Female	Reference					
Male	0.766	0.464−1.251	.288			
Education level						
Under junior high school	Reference			Reference		
Over high school	1.574	1.354−1.932	**.015**	1.595	1.358−1.988	**.025**
BMI	0.861	0.538−1.377	.532			
Smoking (pack/year)	0.695	0.689−0.901	**.013**	0.666	0.589−0.805	**.003**
Biomass						
No	Reference					
Yes	1.528	0.987−2.145	.152			
Occupational exposure						
No	Reference					
Yes	1.028	0.997−1.145	.752			
Duration of asthma	1.528	1.023−1.859	**.012**			
Pulmonary function						
FEV1	0.681	0.488−0.983	**<.001**	0.701	0.591−0.883	**<.001**
FEV1%pred	0.678	0.541−0.830	**<.001**			
FEV1/FVC	0.525	0.412−0.886	**<.001**			
ACT	1.778	1.216−1.987	**<.001**	1.788	1.526−1.977	**<.001**
ACQ	0.698	0.489−0.889	**<.001**			
Asthma control						
Uncontrolled	Reference			Reference		
Partially controlled	2.458	1.365−3.578	**.012**	2.438	0.989−3.489	.052
Well controlled	5.393	1.648−17.646	**.005**	7.006	1.861−26.378	**.004**
Exacerbations in the past year	1.354	0.527−1.627	.128			
Biochemical indexes						
IgE	1.252	0.891−1.435	.239			
FeNO	0.986	0.963−1.098	.825			
Blood eosinophils	0.976	0.861−1.102	.458			

*Note*: The bold *p* values indicate statistical significance.

Abbreviations: a95% CI, adjusted 95% confidence interval; ACQ, asthma control questionnaire; ACT, asthma control test; aOR, adjusted odds ratio; BMI, body mass index; FeNO, fractionated exhaled nitric oxide; FEV1, forced expiratory volume in one second; FVC, forced vital capacity.

### 3.3 The clinical characteristics of smoking, biomass, or occupational exposure alone for patients with asthma

The patients with biomass alone were older, had higher exacerbations in the past year; a longer duration of asthma; and a lower FEV1, FEV1%pred, FVC, IgE, and FeNO compared with the patients with smoking or occupational exposure alone (*p* < .05). Compared with smoking alone, the patients with occupational exposure alone had a longer duration of asthma and lower FEV1, FEV1%pred, FVC, IgE, FeNO, and ICS dose (*p* < .05) (Table 4).

**Table 4:** The clinical characteristics among biomass, occupational exposure, and smoking alone of asthma patients.

Variables	Smoking alone	Occupational exposure alone	Biomass alone	*p* Values
	(*n* = 48)	(*n* = 87)	(*n* = 63)	
Age (years), (mean ± SD)	48.5 ± 12.6^b^	49.2 ± 10.2^b^	56.6 ± 9.8	**<.001**
Sex, *n* (%)				**<.001**
Male	46 (95.8)^a^^,^ b	31 (35.6)^b^	6 (9.5)	
Female	2 (4.2)^a^^,^ b	56 (64.4)^b^	57 (90.5)	
BMI (kg/m^2^), (mean ± SD)	25.3 ± 3.1^b^	24.3 ± 3.7	23.6 ± 3.4	**.042**
Education, *n* (%)				**<.001**
Under junior high school	25 (52.1)^b^	49 (56.3)^b^	55 (87.3)	
Over high school	23 (47.9)^b^	38 (43.7)^b^	8 (12.7)	
Duration of asthma (years), (mean ± SD)	12.7 ± 9.6^a^^,^ b	19.5 ± 11.1^b^	26.7 ± 9.5	**<.001**
Pulmonary function, (mean ± SD)				
FEV1	2.5 ± 0.8^a^^,^ b	2.2 ± 0.8^b^	1.7 ± 0.7	**<.001**
FEV1%pred	86.9 ± 20.7^a^^,^ b	83.0 ± 20.9^b^	77.5 ± 22.4	**<.001**
FEV1/FVC	74.7 ± 11.8^a^^,^ b	67.2 ± 12.0^b^	64.4 ± 13.8	**<.001**
ACT, (mean ± SD)	18.1 ± 4.4	17.4 ± 4.5	17.3 ± 4.6	.636
ACQ, (mean ± SD)	1.0 ± 0.7	1.0 ± 0.7	1.1 ± 0.7	.712
Asthma control, *n* (%)				.151
Well controlled	5 (10.4)	7 (8.0)	4 (6.3)	
Partially controlled	13 (27.1)	27 (31.0)	30 (47.6)	
Uncontrolled	30 (62.5)	53 (60.9)	29 (46.1)	
Exacerbations in the past year, (median, IQR)	0 (0−0)^a^^,^ b	0 (0−1)^b^	0 (0−2)	**.023**
Exacerbations in the past year, *n* (%)				**.005**
0	37 (77.1)^b^	65 (74.7)^b^	33 (52.4)	
≥1	11 (22.9)^b^	22 (25.3)^b^	30 (47.6)	
Biochemical indexes, (median, IQR)				
IgE (mg/L)	342.2 (112.7−650.5)^a^^,^ b	230.4 (123.5−430.6)^b^	153.4 (89.5−438.1)	**.013**
FeNO (ppb)	53.5 (34.8−76.2)^a^^,^ b	45.0 (21.5−64.0)^b^	35.5 (18.0−57.5)	**.017**
Blood eosinophils (×10^9^)	0.5 (0.3−0.8)	0.4 (0.2−0.7)	0.5 (0.3−0.7)	.567
Treatment				
ICS dose (μg/day), (mean ± SD)	321.9 ± 140.6^a^^,^ b	254.7 ± 137.2	257.0 ± 140.3	**.018**

*Note*: The bold *p* values indicate statistical significance.

Abbreviations: ACQ, asthma control questionnaire; ACT, asthma control test; BMI, body mass index; FeNO, fractionated exhaled nitric oxide; FEV1, forced expiratory volume in one second; FVC, forced vital capacity; ICS, inhaled corticosteroids; IQR, interquartile range; SD, standard deviation.

a *p* < .05 versus occupational exposure alone group;

^b^ *p* < .05 versus biomass alone group.

After controlling for potential confounders including sex, age, education level, BMI, and FEV1/FVC. The generalized linear mixed model showed that the biomass alone group had a longer duration of asthma; higher exacerbations in the past year; and lower FEV1, IgE, and FeNO compared with the smoking and occupational exposure alone group. In addition, the occupational exposure alone group had a lower FEV1, FEV1%pred, IgE, and FeNO compared with the smoking exposure alone group (*p* < .05) (Table 5).

**Table 5:** Linear mixed models for the association among asthma patients exposed to smoking alone, biomass alone, and occupational exposure alone.

Variables	Biomass alone versus smoking alone		Biomass alone versus occupational exposure alone		Smoking alone versus occupational exposure alone	
	OR (95% CI)	*p* Values	OR (95% CI)	*p* Values	OR (95% CI)	*p* Values
Duration of asthma	3.562 (1.239−7.593)	**<.001**	1.258 (1.023−1.839)	**.043**	0.723 (0.593−1.125)	.878
Pulmonary function						
FEV1	0.897 (0.563−0.986)	**.015**	0.932 (0.872−0.996)	**.036**	1.258 (1.028−1.935)	**.036**
FEV1%pred	0.798 (0.638−0.896)	**.046**	0.986 (0.966−1.025)	.125	1.326 (1.039−1.995)	**.023**
Exacerbations in the past year	1.531 (1.028−1.908)	**.002**	1.238 (1.012−1.668)	**.012**	0.998 (0.996−1.002)	.485
Biochemical indexes						
IgE	0.785 (0.358−0.982)	**.028**	0.898 (0.568−0.966)	**.041**	2.358 (1.326−3.208)	**.002**
FeNO	0.875 (0.658−0.998)	**.030**	0.736 (0.639−0.838)	**.012**	1.639 (1.056−2.987)	**.003**

*Note*: After adjusted for sex, age, education level, BMI, and FEV1/FVC. The bold *p* values indicate statistical significance.

Abbreviations: BMI, body mass index; CI, confidence intervals; FeNO, fractionated exhaled nitric oxide; FEV1, forced expiratory volume in one second; FVC, forced vital capacity; OR, odds ratio.

### 3.4 The clinical characteristics of never smoking, biomass, or occupational exposure alone for patients with asthma

Compared with the never smoking alone group, the biomass, and occupational exposure alone groups had a higher exacerbation in the past year and a longer duration of asthma, while lower FEV1, FEV1%pred, and FEV1/FVC (*p* < .05) (Supporting Information: Table 1).

After controlling for potential confounders including sex, age, education level, and FEV1/FVC. The generalized linear mixed model showed that the biomass and occupational exposure alone groups had a longer duration of asthma, higher exacerbations in the past year, and lower FEV1 and FEV1%pred compared with the never smoking alone group (*p* < .05) (Supporting Information: Table 2).

## 4 DISCUSSION

Environmental risk factors including smoking, noxious chemicals, occupational exposure, and air pollution are triggers for asthma, especially in adults. In addition, they can lead to a poor outcome, including higher future exacerbations, mortality risk, and persistent airflow limitation.^17^, ^18^, ^19^ In fact, according to the GINA guidelines, the nonpharmacological interventions for patients with asthma include smoking cessation and avoidance of occupational exposure and indoor air pollution are important.^20^

Smoking is the main risk factors for asthma in adults, especially in male. In this study, current and former smokers had a longer duration of asthma; lower ACT sores, FEV1, FEV1%pred, and FVC; and higher ACQ scores compared with never smokers. In addition, current smokers had higher exacerbations in the past year. Several studies have found that smoking contributes to increase the exacerbation risk and symptoms in patients with asthma.^21^ In addition, smoking can lead to an accelerated decline in pulmonary function and increased severity of airflow obstruction.^22^ In this study, we also found that smokers with asthma had worse pulmonary function. FeNO and IgE are both important biomarkers of airway eosinophilic inflammation. Blood eosinophils and FeNO to have comparable diagnostic accuracy which was superior to total serum IgE in adult asthma patients.^23^ In fact, there was study showed that severe asthma with 10‐pack/year history was associated with higher proportion of eosinophilic airway inflammation and autoimmunity toward eosinophils.^24^ Our study found that smoking patients with asthma had a higher value of FeNO, IgE, and blood eosinophils. Long‐term smoking significantly reduces the sensitivity of patients with asthma to ICS: they require higher doses for treatment.^4^ Consistently, we found that patients with asthma had a higher ICS dose upon enrollment.

Of course, quitting smoking is necessary to manage and prevent asthma. Research is needed to explore the factors related to successful smoking cessation so that clinicians can effectively guide patients with asthma to quit smoking. We identified several factors for successful smoking cessation including age, education level, ACT level, and well controlled asthma. Our findings are consistent with previous studies.^25^, ^26^, ^27^, ^28^

In developing counties, most energy for homes is supplied from the biomass, which can be a trigger for asthma.^29^ However, researchers have confirmed that patients with chronic obstructive pulmonary disease (COPD) exposed to biomass have worse pulmonary function compared with those subjected to smoking and occupational exposure.^15^, ^30^ We also found that patients with asthma had worse pulmonary function. In addition, patients with asthma exposed to biomass had lower levels of inflammatory biomarkers, a phenomenon that has also been seen in patients with COPD.^31^

There are some limitations of this study. First, we only examined patients from a single center. Future studies should involve more centers and patients. Finally, we did not stratify the patients according to exposure to different environmental risk factors. This approach would have provided additional information.

## 5 CONCLUSIONS

We identified significant clinical differences among patients with asthma depending on the smoking status. In addition, we noted significant clinical differences among patients with asthma subjected to smoking, biomass, and occupational exposure. The information provided here can help guide clinicians regarding the effects of these risk factors and to encourage them to take effective action to improve the targeted prevention of asthma.

## AUTHOR CONTRIBUTIONS

Laiheng Luo made substantial contributions to conception and design, acquisition of data, or analysis of data; drafted the article; agreed to submit to the current journal; gave final approval of the version to be published and agree to be accountable for all aspects of the work.

## CONFLICT OF INTEREST STATEMENT

The authors declare no conflict of interest.

## ETHICS STATEMENT

This study was conducted in accordance with the Declaration of Helsinki and approved by the Ethics Committee of the Jiangxi Province Hospital of Integrated Chinese and Western Medicine (Number: 202301). All patients provided their informed consent.

## Supporting information

Supporting information.

## Data Availability

The data used and analyzed in this study are available from the corresponding author on reasonable request.
