# Supplementary material for: The clinical characteristics of patients with asthma exposed to different environmental risk factors: A cross‐sectional study
Source: Immun Inflamm Dis. 2023 Jun 28;11(6):e923. doi: 10.1002/iid3.923 (PMC10303691; doi:10.1002/iid3.923)
Supplement: Supplementary file 1 — Supporting information. [file IID3-11-e923-s001.docx]

**Supplemental table 1. The clinical characteristics among biomass, occupational exposure and never smoking alone of asthma patients.**

| **Variables** | **Never smoking**  **alone**  **(n = 208)** | **Occupational exposure alone**  **(n = 87)** | **Biomass alone**  **(n = 63)** | **P-**  **values** |
| --- | --- | --- | --- | --- |
| Age (years), (Mean ± SD) | 46.5 ± 14.3 **^#^** | 49.2 ± 10.2 **^#^** | 56.6 ± 9.8 | **<0.001** |
| Sex, n (%) |  |  |  | **<0.001** |
| Male | 170 (81.7) **^*, #^** | 31 (35.6) **^#^** | 6 (9.5) |  |
| Female | 38 (18.3) **^*, #^** | 56 (64.4) **^#^** | 57 (90.5) |  |
| BMI (kg/m^2^), (Mean ± SD) | 23.8 ± 3.7 | 24.3 ± 3.7 | 23.6 ± 3.4 | 0.377 |
| Education, n (%) |  |  |  | **<0.001** |
| Under junior high school | 104 (50.0) **^#^** | 49 (56.3) **^#^** | 55 (87.3) |  |
| Over high school | 104 (50.0) **^#^** | 38 (43.7) **^#^** | 8 (12.7) |  |
| Duration of asthma (years), (Mean ± SD) | 9.8 ± 6.1 **^*, #^** | 19.5 ± 11.1 **^#^** | 26.7 ± 9.5 | **<0.001** |
| Pulmonary function, (Mean ± SD) |  |  |  |  |
| FEV1 | 3.4 ± 0.9 **^*, #^** | 2.2 ± 0.8 **^#^** | 1.7 ± 0.7 | **<0.001** |
| FEV1 %pred | 87.9 ± 20.7 **^*, #^** | 83.0 ± 20.9 **^#^** | 77.5 ± 22.4 | **<0.001** |
| FEV1/FVC | 84.7 ± 13.2 **^*, #^** | 67.2 ± 12.0 **^#^** | 64.4 ± 13.8 | **<0.001** |
| ACT, (Mean ± SD) | 17.8 ± 4.9 | 17.4 ± 4.5 | 17.3 ± 4.6 | 0.632 |
| ACQ, (Mean ± SD) | 0.9 ± 0.7 | 1.0 ± 0.7 | 1.1 ± 0.7 | 0.173 |
| Asthma control, n (%) |  |  |  | 0.143 |
| Well controlled | 14 (6.7) | 7 (8.0) | 4 (6.3) |  |
| Partially controlled | 64 (30.8) | 27 (31.0) | 30 (47.6) |  |
| Uncontrolled | 130 (62.5) | 53 (60.9) | 29 (46.1) |  |
| Exacerbations in the past year, (Median, IQR) | 0 (0, 0) **^*, #^** | 0 (0, 1) **^#^** | 0 (0, 2) | **0.030** |
| Biochemical indexes, (Median, IQR) |  |  |  |  |
| IgE (mg/l) | 256.4 (127.5, 459.7) | 230.4 (123.5, 430.6) | 153.4 (89.5, 438.1) | 0.274 |
| FeNO (ppb) | 38.0 (22.0, 63.0) | 45.0 (21.5, 64.0) | 35.5 (18.0, 57.5) | 0.254 |
| Blood eosinophils (×10^9^) | 0.5 (0.3, 0.7) | 0.4 (0.2, 0.7) | 0.5 (0.3, 0.7) | 0.376 |
| Treatment |  |  |  |  |
| ICS dose (μg/day), (Mean ± SD) | 223.5 ± 128.2 | 254.7 ± 137.2 | 257.0 ± 140.3 | 0.078 |

**Notes:** ***** P<0.05 versus occupational exposure alone group; **^#^** P<0.05 versus biomass alone group. The bold P values indicate statistical significance.

**Abbreviations:** ACQ, Asthma Control Questionnaire; ACT, Asthma Control Test; BMI, Body Mass Index; FEV1, Forced Expiratory Volume in one second; FVC, Forced Vital Capacity; FeNO, Fractionated Exhaled Nitric Oxide; IQR, Interquartile Range; ICS, Inhaled Corticosteroids; SD, [Standard Deviation](javascript:;).

**Supplemental table 2. Linear mixed models for the association among asthma patients exposed to never smoking alone, biomass alone and occupational exposure alone.**

| **Variables** | **Biomass alone vs never smoking alone** | | **Occupational exposure alone vs never smoking alone** | |
| --- | --- | --- | --- | --- |
|  | **OR (95% CI)** | **P-values** | **OR (95% CI)** | **P*-*values** |
| Duration of asthma | 2.661 (1.143-5.494) | **<0.001** | 1.723 (1.113-4.235) | **<0.001** |
| Pulmonary function |  |  |  |  |
| FEV1 | 0.697 (0.463-0.986) | **<0.001** | 0.758 (0.628-0.968) | **0.006** |
| FEV1 %pred | 0.498 (0.335-0.793) | **<0.001** | 0.626 (0.539-0.995) | **0.008** |
| Exacerbations in the past year | 1.731 (1.158-2.237) | **0.001** | 1.298 (1.126-1.805) | **0.031** |

**Notes:** After adjusted for sex, age, education level, and FEV1/FVC. The bold P values indicate statistical significance.

**Abbreviations:** CI, Confidence Intervals; FEV1, Forced Expiratory Volume in one second; FVC, Forced Vital Capacity; OR, Odds Ratio.
